# Continuous intra-sinus bone regeneration after nongrafted sinus lift with a PLLA mesh plate device and dental implant placement in an atrophic posterior maxilla: a case report

**DOI:** 10.1186/s40729-016-0049-z

**Published:** 2016-06-06

**Authors:** Takahiro Kaneko, Satoshi Nakamura, Shunsuke Hino, Norio Horie, Tetsuo Shimoyama

**Affiliations:** Department of Oral and Maxillofacial Surgery, Saitama Medical Center, Saitama Medical University, 1981 Kamoda, Kawagoe, Saitama 350-8550 Japan

**Keywords:** Bone formation, Sinus membrane elevation, HA/PLLA mesh device

## Abstract

**Background:**

Sinus lift is a bone augmentation procedure that improves the alveolar crest height in an atrophic posterior maxilla. However, the regenerated bone volume can vary and generally has a tendency to decrease after sinus operation. This article describes nongrafted maxillary sinus lift using a bioresorbable unsintered hydroxyapatite combined with poly l-lactide (HA/PLLA) mesh plate device and dental implant placement in an atrophic posterior maxilla, after which continuous bone gain was observed around the implant apex during a postoperative follow-up period of 3 years.

**Case presentation:**

A 60-year-old healthy female was referred to our department for dental implant therapy in the right posterior maxilla. Clinical examination revealed that the maxilla was edentulous from the right first premolar to the second molar region. Radiographically, atrophy of the maxillary alveolar ridge in the same tooth site was observed. Sinus membrane elevation and simultaneous implant placement were performed through the lateral approach. HA/PLLA mesh was utilized to maintain space under the elevated sinus membrane and as a fixation device to replace the bone window. Six months later, new bone was generated in the secluded space maintained under the elevated sinus membrane. When observed 42 months after the implant insertion, bone volume around the implant apex had increased in vertical direction under the HA/PLLA mesh plate device, and there was continuous bone formation in the sinus over time.

**Conclusion:**

This nongrafted sinus lift procedure using an HA/PLLA mesh device attained predictable bone formation. Stable membrane elevation by an HA/PLLA device might induce long-term, continuous bone formation in the sinus.

## Background

Maxillary sinus lift is a bone augmentation procedure in the sinus that improves the alveolar crest height in atrophic posterior maxilla by forming new bone in the space created under the elevated sinus membrane. To date, numerous grafting materials have been used as a scaffold for new bone regeneration, including autogenous bone, bone graft substitutes, or their combination [1, 2]. Autogenous bone graft is considered as gold standard among these grafting materials; however, its limitations include morbidity of donor sites, delay of new bone generation, and complications such as material infection and sinusitis [3, 4].

Recent studies have shown that sinus lift by elevating the sinus membrane without grafting materials induces bone formation in the maxillary sinus [5–8]. Nongrafted sinus lift has been accepted as a predictable bone augmentation procedure in atrophic posterior maxilla. In the sinus lift procedure, bone graft and dental implants serve as space holders under the elevated sinus membrane, and new bone is formed in a secluded space under the elevated sinus membrane, according to the principle of guided tissue regeneration [5]. However, positive intra-sinus air pressure associated with respiration exists in the maxillary sinus [9], and this pressure may have a negative effect on bone formation in the sinus, thus inducing bone resorption and pneumatization after sinus augmentation [10]. Indeed, postoperative vertical bone loss is commonly observed in conventional sinus lift with autogenous bone grafting. Several years after surgery, most grafting materials covering the implant apex get resorbed, and the implant apex is exposed to the grafted sinus floor [11]. Therefore, even the presence of physiological stimuli after implant loading may alter the augmented bone volume surrounding implants in the sinus. It is difficult to maintain postoperative augmented space stable for long term, with or without grafting materials.

This article describes a case of nongrafted maxillary sinus lift using a mesh plate device consisting of bioresorbable unsintered hydroxyapatite combined with poly l-lactide (HA/PLLA) materials and dental implant placement in an atrophic posterior maxilla. In this case, continuous bone formation in the sinus was observed during the postoperative follow-up period of 3 years, and we show the sequential radiographic outcome of this procedure.

## Case presentation

**Fig. 1:**
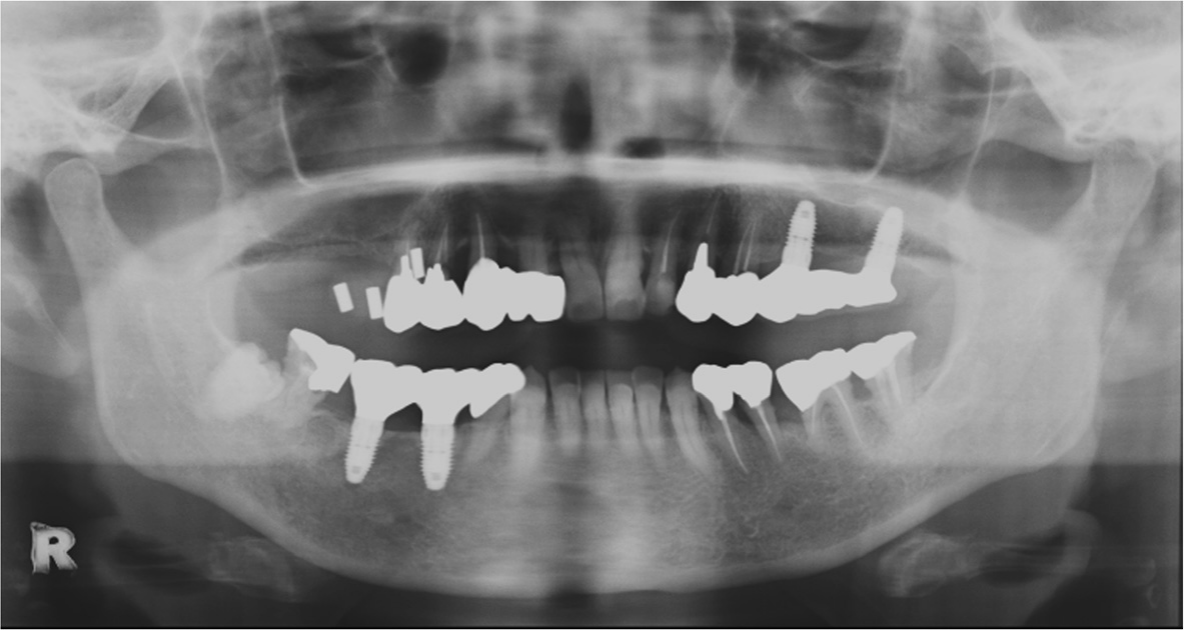
Preoperative panoramic radiograph finding

A 60-year-old healthy female, who desired dental implant therapy in the right molar region of the maxilla, was referred to the Department of Oral and Maxillofacial Surgery. Clinical examination revealed an edentulous maxilla from the right first premolar to the second molar region. Panoramic radiography showed atrophy of the maxillary alveolar ridge in the same site (Fig. 1), and the need for sinus augmentation was confirmed by computed tomography (CT). The patient was informed of the details of the treatment protocol, in particular the sinus surgery, and she consented to participate. A written consent was obtained.

**Fig. 2:**
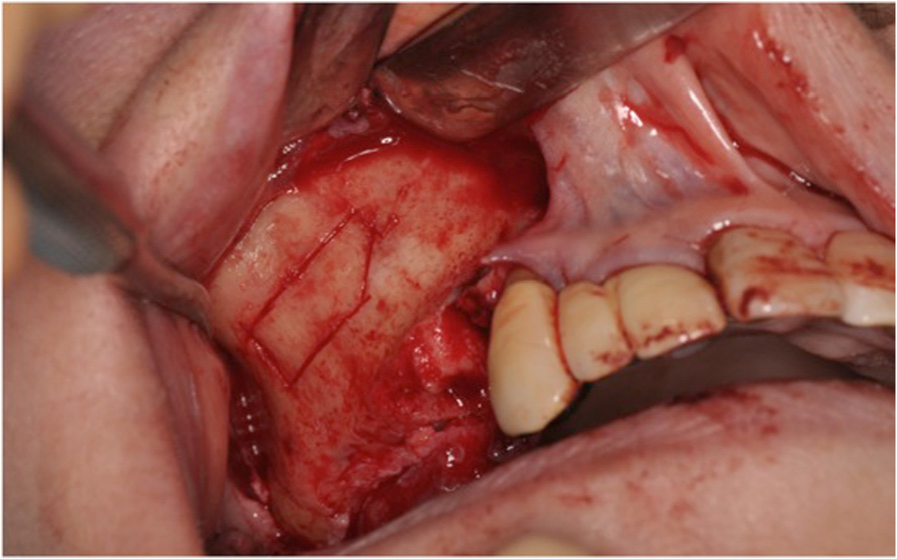
A trapezoidal bone window was made in the lateral sinus wall by osteotomy

**Fig. 3:**
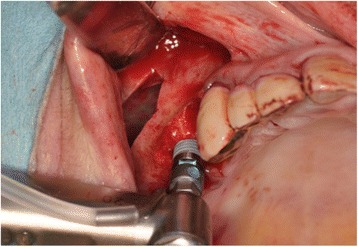
After the removal of the bone window, the membrane was lifted upward, and the dental implants were placed without grafting materials

**Fig. 4:**
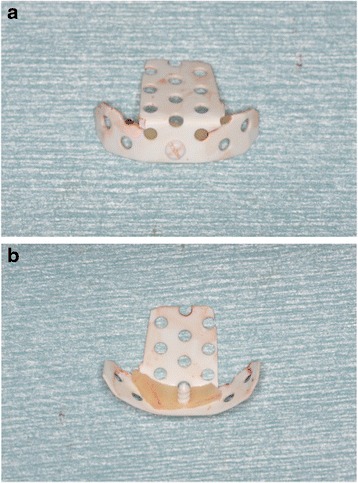
**a** Front view of the bone window with the HA/PLLA mesh plate device fixed by a screw. **b** Lower view of the bone window with the HA/PLLA mesh plate device fixed by a screw

**Fig. 5:**
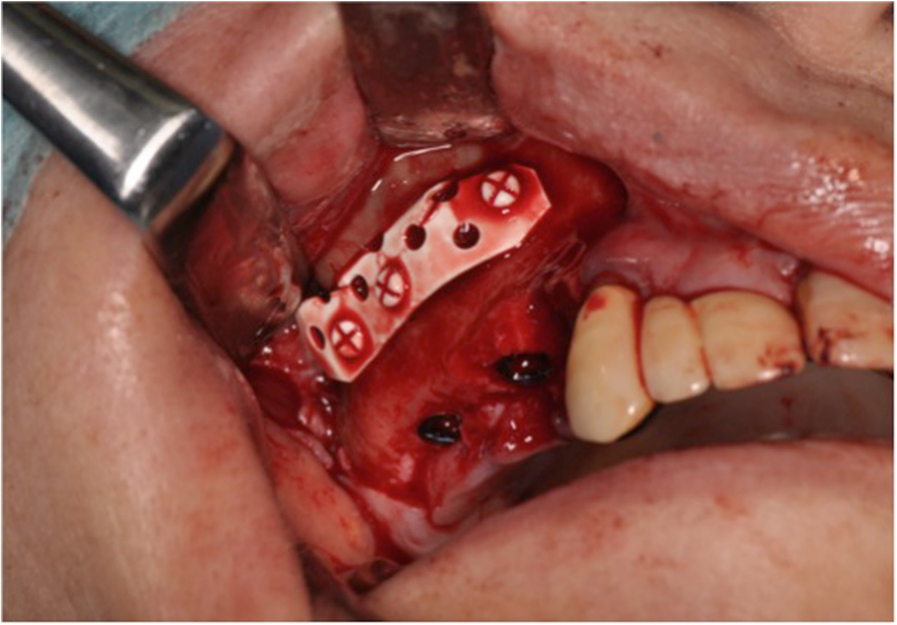
Clinical view of the repositioned bone window with the HA/PLLA mesh plate device

**Fig. 6:**
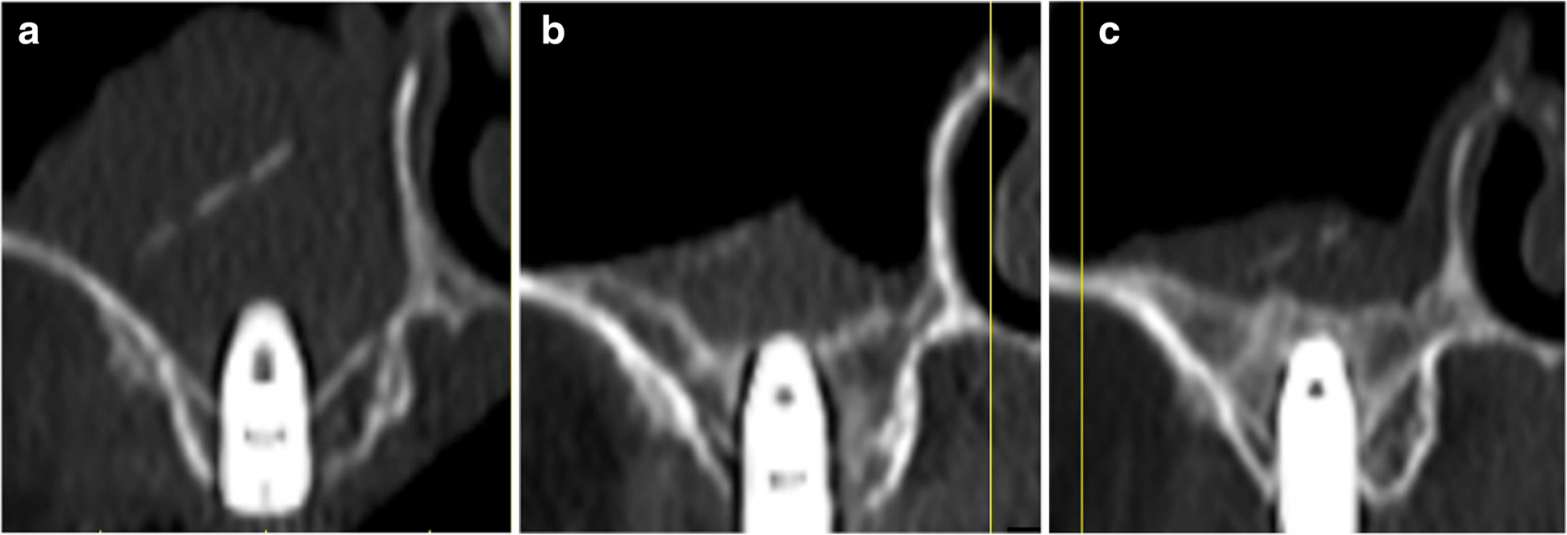
Radiographic findings on cross-sectional computed tomography in the right second molar region after nongrafted sinus lift with simultaneous implant placement. **a** Immediately. **b** At 6 months. **c** At 42 months

**Fig. 7:**
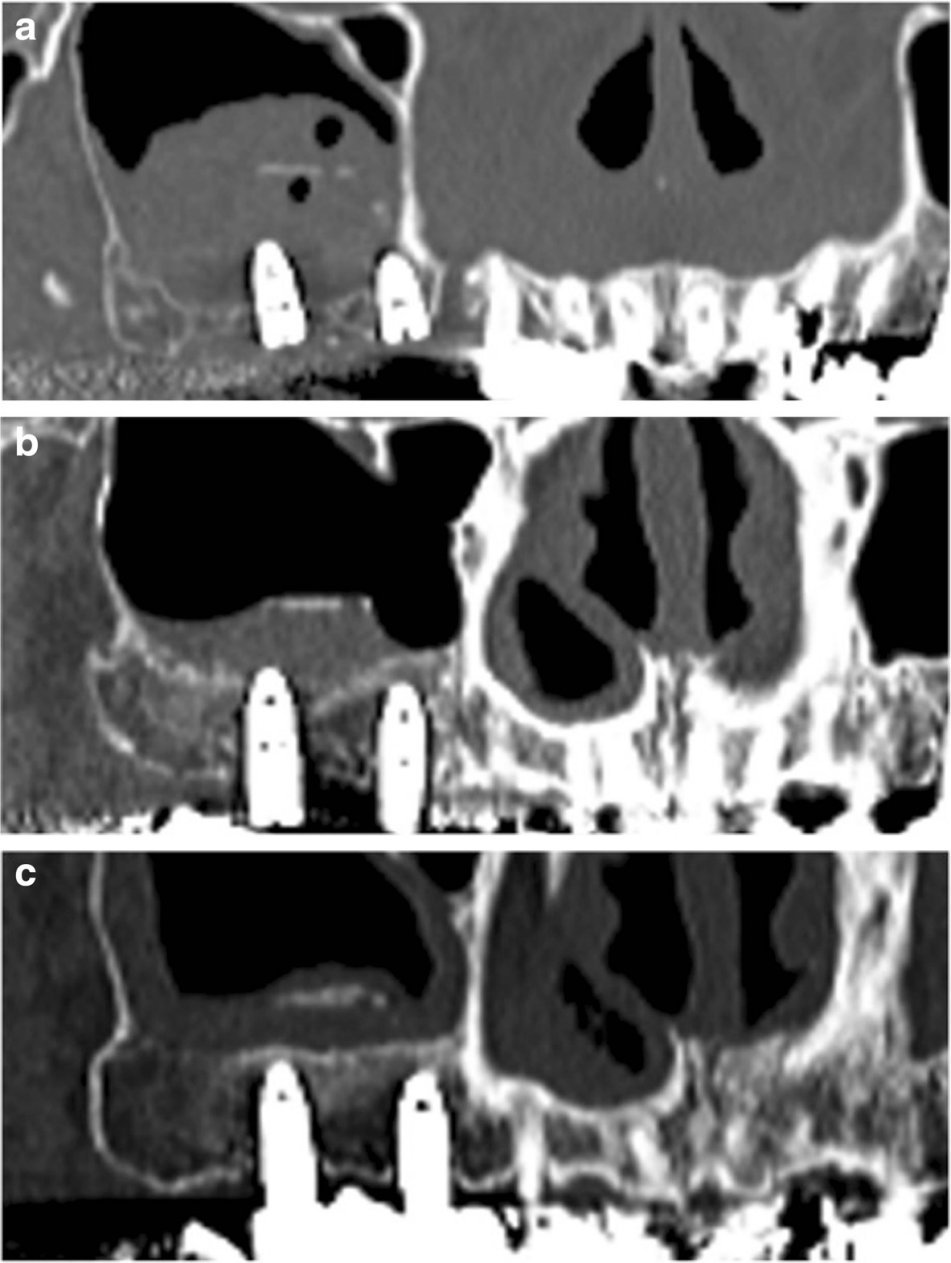
Radiographic findings on panoramic computed tomography after nongrafted sinus lift with simultaneous implant placement. **a** Immediately. **b** At 6 months. **c** At 42 months

**Fig. 8:**
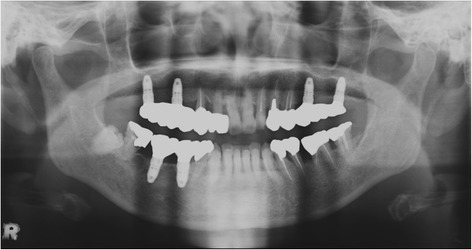
Postoperative panoramic radiograph taken about 3 years after implant loading

Maxillary sinus lift and simultaneous implant placement were implemented by sinus membrane elevation using an HA/PLLA mesh device without grafting materials. Using a piezoelectric device (Piezosurgery®; Mectron Medical Technology, Carasco, Italy), osteotomy was performed in the lateral sinus wall of the right posterior maxilla (Fig. 2). After the removal of the bone window, the membrane was carefully elevated from the sinus floor and two implants (Replace Select™ Tapered, TiUnite™; Nobel Biocare AB, Göteborg, Sweden) were inserted through the maxillary ridge into the space created under the elevated sinus membrane (Fig. 3). Subsequently, the bone window was replaced and measures for maintaining the space under the elevated sinus membrane were taken using an HA/PLLA mesh plate device (Super FIXSORB®-MX, Takiron Co., Ltd., Kobe, Japan). To support the membrane, the device was bent in L form in warm water at 75 °C and attached to the bone window (Fig. 4a, b). Using the mesh plate, the sinus membrane was lifted, and bone window was fixed to the sinus wall using two short HA/PLLA screws (Fig. 5). The abutment was connected 6 months after the sinus surgery. During the follow-up periods after the first and second surgeries, wound healing was uneventful and no intra-sinus problem was observed. Four months after the abutment connection, screw-retained implant prosthesis was fabricated and loaded. Pre- and postoperative alveolar crest heights were evaluated using CT reformatted using Simplant Pro™ software (Materialise Dental NV, Leuven, Belgium) (Figs. 6a–c and 7a–c). Postoperative radiographs were taken immediately and at 6 and 42 months following implant insertion. Six months after implant insertion, the implant was entirely embedded in the newly formed bone under the HA/PLLA mesh device surrounded by soft tissue density (Figs. 6b and 7b). Additional bone formation in vertical direction was noted in the space under the HA/PLLA mesh plate device 6 to 42 months after implant insertion. At 42 months after implant insertion, continuous increase of bone volume above the implant apex was confirmed without excessive marginal bone loss around the implant neck (Figs. 6c and 7c). It has now been more than 3 years since the installation of the permanent restoration, and no problems have been observed (Fig. 8).

### Discussion

In nongrafted sinus-lifting procedure, several devices such as titanium [12–14], hollow hydroxyapatite [15], and bioresorbable materials [16] have been used for space retention to maintain the lifted sinus membrane and the results of predictable bone formation have been reported in addition to histological examination. In this case, a mesh plate device consisting of HA/PLLA materials was applied for space maintenance under the elevated sinus membrane in combination with dental implant placement. PLLA materials are generally used for bone fixation in the extremities and maxillofacial region [17]. Since the PLLA materials can stay firm and stable in vivo for several years, it probably has enough strength to resist air pressure in the sinus. In addition, the flat surface of the mesh device could provide wide elevation of the sinus membrane and provides a favorable condition to facilitate bone formation in nongrafted sinus lift procedure.

In conventional sinus lift with grafting materials, the materials are usually transplanted to the secluded space under the lifted sinus membrane covering the entire length of the implants [1], and in the initial stage after sinus augmentation, the grafted sinus floor is consistently located above the implant apex [11]. However, the augmented bone volume has a tendency to decrease postoperatively. After 2–3 years, it would generally reach the level same as or slightly below the implant apex [11]. In addition, such tendency for bone loss seems to be consistent in the sinus lift procedure without grafting materials [18] and might reflect the influence of physiological stimuli by implant loading and maxillary sinus ventilation [11]. Conversely, a few studies on nongrafted sinus lift procedure showed a contentious increment of intra-sinus bone volume [7, 13]. Nedir et al. [7], in their sinus lift procedure using the crestal approach without grafting materials, reported postoperative bone gain around the implant apex that increased slightly over the course of 2 years after surgery. In our case, the bone regeneration surrounding the protruded implant was confirmed at 6 months after implant insertion; thereafter, during a follow-up period of about 3 years, additional increase of new bone occurred above the implant apex and new bone was continuously formed in the sinus. In the follow-up CT taken at 42 months after the implant insertion, there was no significant change in the situation of the HA/PLLA mesh device 6 months after the implant insertion and the condition of lifted sinus membrane was stable. Therefore, the intra-sinus augmented site under the HA/PLLA mesh plate was not affected by pneumatization due to air pressure associated with respiration, which could contribute to the maintenance of bone volume after sinus lift [11]. In nongrafted sinus lift, blood clots are the only filling material around the inserted implants, and the recruitment and migration of osteogenic cells to the blood clot that fills the defect under the elevated sinus membrane generally occur from the bone marrow of the alveolar bone, the periosteum of the elevated sinus membrane, or both [18–20]. Recent studies demonstrated that the maxillary Schneiderian membrane presents osteogenic potential and contributes to the process of bone regeneration, as evidenced by in vivo and in vitro studies [19, 20]. Interestingly, in our case, bone formed from the intra-sinus dense soft tissue under the HA/PLLA mesh plate device. Soft tissue density could be regarded as postoperative hyperplasia of sinus membrane. Therefore, it may reflect the osteogenic property of the maxillary Schneiderian membrane, and new bone deposition induced by the migration of osteogenic cells originating from the Schneiderian membrane might improve vertical bone height around the implant apex. Whereas although PLLA materials are biodegradable and complete degradation in vivo normally occurs over 5 years [17], intra-sinus air pressure could affect the augmented sites again after degradation of materials, and bone loss above implant apex and pneumatization may occur in the sinus thereafter. In addition, several clinical studies of orthognathic and fracture surgery in the maxillofacial unit using PLLA device reported adverse events such as wound infection after surgery [17]. Therefore, prudent observation is required to prevent the development of complications such as sinusitis or implantitis over the long term.

## Conclusions

This nongrafted sinus-lifting procedure using an HA/PLLA mesh plate device helps to attain predictable bone formation. Stable membrane elevation by the HA/PLLA device for the long term could contribute to predictable bone formation in the sinus. The source of cell supply could possibly be the Schneiderian membrane, reflecting its osteogenic potential.

## Consent

Written informed consent was obtained from the patient for publication of this report and all accompanying images.

## Abbreviations

CT: computed tomography
HA/PLLA: bioresorbable unsintered hydroxyapatite combined with poly l-lactide
